# The Effect of Electroacupuncture Treatment with Different Intensities for Functional Diarrhea: A Randomized Controlled Trial

**DOI:** 10.1155/2022/2564979

**Published:** 2022-01-04

**Authors:** Xiaohu Xu, Mingmin Zhang, Xiao Wu, Cuihong Zheng, Guangying Huang

**Affiliations:** ^1^Department of Integrated Traditional Chinese and Western Medicine, Tongji Hospital, Tongji Medical College, Huazhong University of Science and Technology, Wuhan 430030, China; ^2^Institute of Integrated Traditional Chinese and Western Medicine, Tongji Hospital, Tongji Medical College, Huazhong University of Science and Technology, Wuhan 430030, China

## Abstract

**Background:**

Electroacupuncture (EA) may have a role in the treatment of diarrhea symptoms. However, the efficacy and safety of EA with different current intensities in improving gastrointestinal function, psychology, and quality of life (QOL) of functional diarrhea (FD) remain unknown.

**Objective:**

To investigate the efficacy and safety of EA with different current intensities in improving gastrointestinal function, psychology, and QOL for FD patients.

**Methods:**

73 FD patients were randomly divided into three groups: low current intensity group (LI) of EA, high current intensity group (HI) of EA, and loperamide control group (LC). Four weeks of treatment were provided in the three groups. The primary outcome was the proportion of normal defecation. Additional outcomes included the change from baseline for the weekly spontaneous bowel movements (SBMs) and the change from baseline for the mean Bristol Stool Form Scale (BSFS). QOL was assessed by the 36-item short-form health survey (SF-36). Self-rating Anxiety Scale (SAS) and Self-rating Depression Scale (SDS) were used to assess the psychology state.

**Results:**

Low current intensity of EA significantly improved the proportion of normal defecation during treatment and follow-up period (*P* < 0.01). EA significantly improves the mean BSFS scores and weekly SBMs, and this efficacy is equivalent to loperamide (*P* < 0.05). The SF-36 scores of general health in LI and HI groups and vitality and mental health in LI group were significantly increased compared to baseline (*P* < 0.05). Low current intensity of EA can significantly improve SAS and SDS scores (*P* < 0.05).

**Conclusions:**

EA significantly improved stool consistency and weekly SBMs in FD patients. Compared with loperamide, low current intensity of EA may have a better sustainable effect in restoring normal defecation in patients with FD, and it can also effectively improve QOL, anxiety, and depression. However, larger sample sizes are needed to determine safety and efficacy. Trial registration number: NCT01274793.

## 1. Introduction

Diarrhea is an intestinal disorder characterized by fluidity of fecal evacuations and abnormal frequency. Patients with diarrhea lasting more than 4 weeks are usually diagnosed with chronic diarrhea. Irritable bowel syndrome (IBS) and functional diarrhea (FD) are the most common types of chronic diarrhea. FD is a continuous or recurrent syndrome characterized by the passage of loose (mushy) or watery stools without abdominal pain or discomfort, which distinguishes it from IBS with diarrhea (IBS-D) [1]. The estimated prevalence of FD is 6.0% in the USA and 1.54% in China [1,2]. The Rome III diagnostic criteria require loose (mushy) or watery stools without pain with at least 75% of bowel movements for the last three months with symptom onset at least six months before diagnosis [3]. The pathogenesis of FD is still unclear, but may be related to intestinal dysfunction, destruction of the mucosal barrier, gastrointestinal motility disorders, visceral hypersensitivity, diet, and psychological and genetic factors [4]. Although the prognosis of FD is not bad, it places a considerable medical, social, and economic burden on individuals and societies. The conventional medication for FD is antidiarrheal therapy, such as diphenoxylate or loperamide, which provides symptomatic relief. However, a significant proportion of patients may stop responding to conventional medication and be affected by adverse effects such as constipation and bloating. Therefore, nonpharmacological treatments including complementary and alternative medicine (CAM) are often used in order to alleviate these problems.

Traditional Chinese Medicine (TCM), as one of CAM therapies, has been used for the treatment of gastrointestinal diseases for more than 3000 years in history. It has been found that it can improve gastrointestinal symptoms, and the feedback from patients is good. Among the TCM treatments, acupuncture is a characteristic external therapy that has been used to ameliorate diarrhea [5]. According to TCM theory, the most popular treatment targets in acupuncture treatment of diarrhea are traditional acupuncture points ST25 (*Tianshu*), BL25 (*Dachangshu*), ST37 (*Shangjuxu*), and ST36 (*Zusanli*). In recent years, a large number of clinical and animal studies have shown that the influence of acupuncture on gastrointestinal motility is related to autonomic nerve reflex and gut-brain axis [6]. For example, one study demonstrated that acupuncture at ST25 modulates gastrointestinal motility, increases the threshold of visceral sensitivity, and regulates gastrointestinal hormones [6]. Another study has indicated that acupuncture at ST25 can slow gastrointestinal motility by activating *β*1/2 receptors [7]. A further study showed that acupuncture at ST36 increases vagal activity, resulting in potentiation of the accommodation reflex [8]. Electroacupuncture (EA) has the advantage of providing controllable current frequency and intensity stimulation, which is widely used in research and clinical practice. Many researches have shown that acupuncture is an effective method to treat functional gastrointestinal disorders (FGIDs) [9]. For example, our previous studies have shown that EA is effective and safe for functional constipation [10,11]. However, the effect of electroacupuncture on FD has been rarely studied. Acupuncture has been applied at different traditional acupuncture points or stimulation frequencies in different studies [12–14], trials conducted using different current intensities of EA for FD are infrequent, and whether varying these parameters can strengthen the therapeutic effect of EA remains unknown.

Moreover, according to the research conducted in the United States, FD is also related to the high incidence and severity of depression [15]. This study evaluated diarrhea in the general population and found that depression is an important predictor of chronic diarrhea. Similarly, patients diagnosed with depression report significantly more frequent gastrointestinal symptoms compared to individuals without depression, with bowel-related symptoms highly associated with depression severity scores [16]. Although the comorbidity etiology of FD and depression remains unclear, one prevalent theory is that the symptoms result from a disturbance in neurotransmitter-related regulation of communication between the enteric nervous system and the brain, or called “the brain–gut axis.” To the best of our knowledge, the effectiveness of EA on the psychology of FD patients has not been reported. For these reasons, we designed a randomized controlled trial (RCT) using EA at ST25 and BL25 with different current intensities and a fixed frequency of 2/50 Hz for FD. The purpose of this study was to investigate the effects and safety of EA with different current intensities on gastrointestinal function, psychology, and quality of life (QOL) in patients with FD.

## 2. Methods

### 2.1. Study Design

This study was a RCT. The total study period was nine weeks, comprising a 1-week baseline assessment, 4-week treatment, and 4-week follow-up. The Clinical Trial Ethics Committee of Tongji Medical College, Huazhong University of Science and Technology, approved our study design; approval document number is FWA00007304. This study has been registered in ClinicalTrials.gov (NCT01274793) and conducted according to the Helsinki Declaration and Good Clinical Practice Guidelines.

### 2.2. Patients

We included patients who (1) met the requirements of the Rome III diagnostic criteria for functional diarrhea; (2) were aged 18 to 65 years; (3) had not used any drugs that may affect gastrointestinal motility or secretion for at least one week prior to randomization; (4) did not participate in any other trial in progress; (5) signed the informed consent form voluntarily; and (6) were able to complete the whole study. Patients were excluded due to the following conditions: (1) diarrhea caused by other diseases or drugs; (2) structural, inflammatory gastrointestinal disease or IBS; (3) mental illness, cognitive impairment, or aphasia; (4) tumours and bleeding; (5) severe heart, liver, kidney disease; and (6) pregnancy, lactation, and other serious diseases that may affect the completion of the study.

Recruitment advertisements in newspaper and the hospital website (http://www.tjh.com.cn/) were used to help recruit patients. We recruited patients from the following hospitals: Tongji Hospital, Tongji Medical College, Huazhong University of Science and Technology (HUST) and Hospital of Huazhong University of Science and Technology. The first patient was enrolled on 14 December, 2011, and the last patient completed the follow-up period on 29 May, 2015.

Written informed consent of all patients is required before participating in this study. They were also required to have an electrocardiogram (ECG) and colonoscopy examination. Urine, stool, and blood biochemical tests were performed before and after treatment, including alanine aminotransferase (ALT), aspartate aminotransferase (AST), blood urea nitrogen (BUN), and serum creatinine. Patients received 1-week baseline evaluation before being randomized, and entered another 4-week follow-up period after treatment. Throughout the whole study period, patients recorded stool consistency, frequency of defecation, and adverse events every day.

### 2.3. Randomization and Blinding

According to the ratio of 1 : 1 : 1 using random number table method, patients were randomized divided into three groups: low current intensity group (LI) of EA, high current intensity group (HI) of EA, and loperamide control group (LC). The randomization sequence was generated using R2.0 software. A designated researcher prepared the assignments in opaque envelopes in sequence. One person at each hospital was responsible for the envelopes. The acupuncturists only knew of the group assignment immediately prior to the treatment. Patients, outcome assessors, and statisticians were all unaware of treatment allocations. EA will be manipulated by an experienced acupuncturist. An independent researcher in Tongji hospital evaluated all the collected outcome data. In order to ensure consistency, all researchers accepted standardized professional training before the implementation of the research.

### 2.4. Interventions

The acupuncture treatment was based on TCM theory and provided by licensed acupuncturists. Treatment was administered bilaterally at ST25 and BL25, which are very commonly utilised in FD patients. After the skin was prepped with alcohol, 0.30 × 40 mm or 0.30 × 50 mm sterile disposable acupuncture needles (Human Health, Shanghai, China) were inserted at ST25 and BL25. The acupuncturist rotated the needle to create a *de qi* sensation that included soreness, numbness, heaviness, and distension. 0.18 × 13 mm auxiliary needles (Human Health, Shanghai, China) were inserted 2 mm away from ST25 or BL25 point locations, with a vertical depth of 2 mm, without manual stimulation. Then, each needle was connected to HANS-200E electroacupuncture instrument (Jisheng, Nanjing, Jiangsu, China) and stimulated by electrical stimulation for 30 mins at 2/50 Hz. For the LI group, the stimulation intensity varied from 0.1 mA to 0.8 mA, which was weak, but patient could feel it. For the HI group, the stimulation intensity varied from 1.0 mA to 1.8 mA, which was strong enough, but patient can tolerate it. Patients received 16 times of EA treatment, 5 times/week in the first two weeks, and 3 times/week in the next two weeks. For the LC group, patients were given loperamide hydrochloride capsule 2 mg (Xi'an Janssen Pharmaceutical Co., Ltd., Shaanxi, China), 3 times/day for 4 weeks.

### 2.5. Assessments

The primary outcome of the study was the proportion of FD patients with normal defecation, defined as the proportion of a daily stool frequency ≤3 times and stool consistency to be type 4. Additional outcomes included the change from baseline in weekly spontaneous bowel movements (SBMs) and the change from baseline for mean Bristol Stool Form Scale (BSFS) scores. QOL was evaluated by the 36-item short-form health survey (SF-36) [17]. The psychological state of participants was measured with Self-rating Anxiety Scale (SAS) and Self-Rating Depression Scale (SDS) questionnaires [18].

The weekly SBMs is calculated based on daily records completed by participants. The BSFS was used to evaluate the stool consistency. These outcomes were measured at baseline and at the 2nd, 4^th^, and 8th weeks. Patients were required to complete the SF-36 at baseline and at the 4th week. The SAS and SDS were completed at baseline and at the 2nd and 4th weeks. Adverse events during the study period were also evaluated.

### 2.6. Sample Size

We estimate the sample size based on a previous study [19] and calculate it according to the sample content formula compared with multiple sample means:

(1) $$\begin{array}{c} n=\frac{\psi^{2}\left(\sum_{j=1}^{k}s_{j}^{2}/k\right)}{\sum_{j=1}^{k}{\left({\bar{x}}_{j}-\bar{\bar{x}}\right)}^{2}/\left(k-1\right)}. \end{array}$$

After calculation, the number of samples required in each group is 69 and is calculated at the rate of 15% loss; there should be no less than 79 cases in each group and 237 cases in all three groups. However, due to difficulty with participant recruitment and budgetary limitations, enrollment was concluded before the sample size we planned was reached.

### 2.7. Statistical Analysis

The SAS statistical package program version 9.2 (SAS Institute, Cary NC, USA) was used. All *P* values were based on two-sided tests. *P* < 0.05 was considered to be a statistically significant difference. Statistical analysis included a full-analysis set (FAS, the main set of therapeutic evaluation and analysis) and safety set (SS, the main set of safety evaluation). Efficacy analysis was based on an intent-to-treat population. Continuous variables were presented as mean ± SD. Categorical variables were expressed using frequencies and percentages unless stated otherwise. Categorical variables were analyzed with the use of the Cochran-Mantel-Haenszel-*χ*2 (CMH-*χ*2) test. Comparison of continuous variables in the baseline period between the three groups was analyzed with analysis of variance (ANOVA). An analysis of covariance (ANCOVA) with fixed-effect terms for study group and center, and with the corresponding baseline value as a covariate, was used for comparisons during treatment and follow-up periods between the three groups. Finally, we used the test of least significant difference (LSD) for further pairwise comparison if there was a statistically significant difference.

## 3. Results

### 3.1. Participant Flow and Demographics

**Figure 1:**
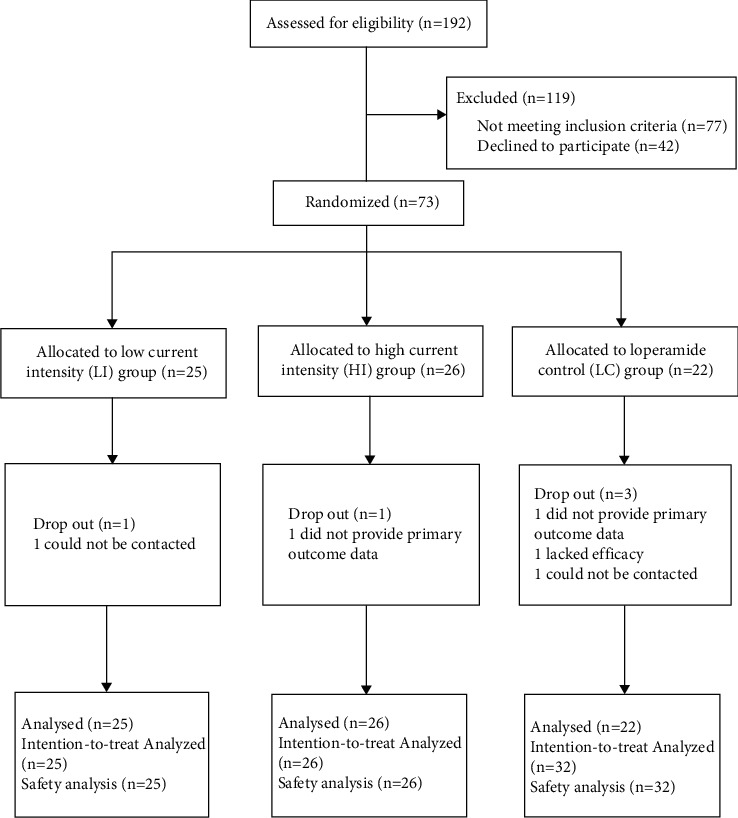
Flow chart of study participants.

Between December (2011) and May (2015), 192 patients with FD were assessed for eligibility. After being screened, 73 patients were randomly assigned to three different groups: low current intensity (LI) group (*n* = 25), high current intensity (HI) group (*n* = 26), and loperamide control (LC) group (*n* = 22) (Figure 1). 5 patients failed to complete the study for various reasons, so 68 patients completed the study. There were no statistically significant differences found in the baseline characteristics among the three groups (Table 1).

### 3.2. Primary Outcome

The primary outcome was the proportion of normal defecation of FD. The change from baseline for the proportion of normal defecation had significantly improved in LI and LC group after treatments (*P* < 0.01), but no significant difference was found between the three groups. This outcome had significantly improved in LI and HI group during follow-up (*P* < 0.01), but no significant difference was found between them. And there was also no significant difference found in LC group during follow-up (Table 2).

### 3.3. Additional Outcomes

The change from baseline for the mean BSFS scores was significantly decreased in three groups (*P* < 0.01) (Table 2). The change from baseline for the mean BSFS score in LC group was significantly improved compared with LI and HI at 2 W (*P* < 0.05), but there is no significant difference for LI and HI compared with LC at 4 W and 8 W (Table 2).

The change from baseline for the weekly SBMs was significantly improved in EA group and LC group at 4 W and 8 W compared to the baseline (*P* < 0.05), but there was no significant difference between these groups (Table 2).

The SF-36 scores of general health in LI and HI groups and vitality and mental health in LI group were significantly improved compared to the baseline phase (*P* < 0.05). No significant improvement in SF-36 scores was found after LC treatment (Table 3).

The change from baseline for the SAS and SDS scores were significantly improved in LI group after treatment (*P* < 0.01), and these effects were better than LC group (*P* < 0.05) (Table 4). There was an significant difference for the SAS in HI group after treatment, while no significant difference was found in LC group for the SAS and SDS scores after treatment (Table 4).

### 3.4. Adverse Events

There were no adverse events reported in the three groups. A total of 5 patients did not complete the study, 2 could not be contacted, 2 did not provide primary outcome data, and 1 lacked efficacy.

## 4. Discussion

Electroacupuncture (EA), as a simple, convenient, quantifiable, and effective therapy, has been widely used for treating many diseases. In the past, most of researches had been spent to compare the effectiveness between manual-acupuncture and electroacupuncture or to demonstrate the effectiveness of different points using electroacupuncture. In fact, the intensity of EA had an influence on its therapeutic effects, which was an important parameter in clinical treatment [20]. In order to standardize and optimize the clinical application of acupuncture, researchers should determine appropriate stimulation intensity to achieve a better therapeutic effect. Most researchers thought that the lower stimulation intensity produced minimal effects, and the higher stimulation intensity had the greater effects [21–23]. However, some clinical and animal experiments revealed the opposite results [24,25]. Therefore, it is necessary to further explore the relationship between stimulation intensity of EA and the therapeutic effect. As far as we know, this is the first randomized controlled trial to compare the efficacy of EA with different current intensities in treating FD patients.

In this study, the efficacy of EA with different current intensities on the change of consistency of stools, weekly SBMs, SF-36, SAS, and SDS for FD patients was assessed after 4 weeks of treatment and 4 weeks of follow-up. As for the theoretical basis of acupoint selection, we have screened acupoints through ancient books and published articles. According to the TCM theory, the Back-Shu and Front-Mu points (the abbreviation for Shu-Mu points), which are located in the lower back or abdomen of the body, are commonly used acupoints to relieve diarrhea symptoms. BL25 (Dachangshu) is the Back-Shu point of large intestine, which is used to treat bloating, diarrhea, and constipation. ST25 (Tianshu) is the Front-Mu point of large intestine, which is used to alleviate abdominal pain, abdominal distension, constipation, diarrhea, dysentery, and other gastrointestinal diseases. Some studies have indicated that EA at ST25 (Tianshu) and BL25 (Dachangshu) was an effective treatment for diarrhea; for example, it could decrease colonic enterochromaffin cell number, colonic tryptophan hydroxylase expression, and 5-HT content in IBS-D rats [26]. Recent studies have also shown that EA at ST25 and BL25 can improve symptoms of FD through reducing expression of 5-HT_1A_R and c-Fos proteins in the hypothalamus and colon tissue in FD rats [27]. It can also reduce intestinal sensitivity of rats with IBS, which may be related to downregulating the expression of M3R and 5-HT_3A_R in the colon [28]. Therefore, we selected ST25 (Tianshu) and BL25 (Dachangshu) acupoints in this trial. Loperamide was frequently used to treat patients with diarrhea. It was found to possess antimotility and antisecretory effects through blocking the *μ*-opioid receptor of the gastrointestinal tract and antagonizing calmodulin [29]. Therefore, we chose loperamide instead of placebo needle as a negative control group, because our purpose was to explore the therapeutic effect of EA at different current intensities on FD and whether EA was superior to conventional treatment.

Both the consistency of stool and defecating frequency were important characteristics reflecting bowel function of FD. In order to better evaluate the effect on bowel function of FD, we chose the proportion of normal defecation of FD as a primary outcome. Our study result showed that low current intensity of EA improved the proportion of normal defecation both in treatment and follow-up period, illustrating a sustained effect in restoring bowel function of FD, while the high current intensity of EA and loperamide did not. Compared with baseline, the mean BSFS scores in all three groups decreased significantly after treatment and follow-up. In addition, EA can also reduce defecation frequency of FD patients, which was similar to loperamide.

The physical, psychological, and social functions of individuals are gradually used as important indicators to evaluate the clinical efficacy of chronic disease [30]. In particular, health-related quality of life has been widely used in the selection of clinical treatment programs, preventive interventions, and clinical medicine, as well as evaluation of preventive medicine, pharmacy, and health management [31,32]. The SF-36 is widely used in the measurement of quality of life for the general population, evaluation of clinical trial results, and health policy assessment. The higher score of each item indicates the better effect on the quality of life. In our trial, the scores of general health in LI and HI groups and vitality and mental health in LI group were significantly increased compared to the baseline phase. However, the effect of loperamide on SF-36 scores is not obvious. It demonstrated that EA had a potential to improve the quality of life of FD.

Recent research had proved that functional gastrointestinal disorder (FGID) patients were involved with anxiety and depression [33]. Psychology or emotional state played an important role in the development of FD [34]. Anxiety and depression might induce FD through the neuro-endocrine-immune network system to influence colonic motor alterations [35]. Our study showed the SAS score was increased after 4 weeks of EA treatment, while the score was not increased after 4 weeks of loperamide treatment. Besides, the low current intensity of EA significant increased SDS score while the high current intensity of EA and loperamide did not. These demonstrated low current intensities of EA showed greater improvement regarding. anxiety and depression.

This study had several limitations. First, the sample size in this trial was small. One of the reasons was most of chronic diarrhea patients who participated in screening were finally diagnosed with irritable bowel syndrome (IBS). Another reason was many patients who met our criteria were not able to finish a frequency of 7 days/week electroacupuncture treatment for 4 weeks. Besides, many participants hesitated to participate in this trial because they were reluctant to assign to drug treatment. Second, this study may suffer from bias, because lots of Chinese had the experience of acupuncture, which may introduce bias to the study results. In addition, it was also not possible to blind acupuncturists to treatment. Outcome measures were not prespecified in the clinicaltrials.gov registration, which is also a potential limitation.

In conclusion, both high and low current intensities of EA significantly improved consistency of stools and weekly SBMs in FD patients without adverse effect; this efficacy was equal to loperamide. The difference between low and high intensity of EA in improving consistency of stools and weekly SBMs was not significant. However, low current intensity of EA has a sustained effect in restoring normal defecation of FD compared with loperamide. In addition, it could effectively improve QOL, anxiety, and depression relative to loperamide. Further studies investigating molecular mechanism implicated in the different intensity EA response are warranted.

## Figures and Tables

**Table 1:** Summary of demographics and baseline patient characteristics.

	LI	HI	LC
*N*	24	25	19
Age, years, mean (SD)	37.69 (16.7)	38.81 (19.0)	41.50 (17.9)

*Gender, no. (%), mean (SD)*			
Female	13 (52.00)	16 (64.00)	8 (40.00)
Male	12 (48.00)	9 (36.00)	12 (60.00)

*Educational level, no. (%), mean (SD)*			
University or above	18 (72.00)	21 (80.00)	16 (80.00)
Secondary or below	7 (28.00)	5 (20.00)	4 (20.00)

*Height, cm, mean (SD)*			
Female	159.42 (5.26)	159.33 (4.56)	160.42 (1.88)
Male	171.00 (6.60)	169.38 (5.54)	170.50 (4.11)

*Weight, kg, mean (SD)*			
Female	53.00 (5.05)	56.00 (5.85)	51.88 (4.29)
Male	63.04 (6.80)	67.06 (10.90)	66.25 (9.50)

Duration of diarrhea, months, mean (SD)	71.56 (91.70)	73.56 (79.04)	60.65 (78.69)

*Pretreatment baseline, median (interquartile)*			
Weekly SBMs	12.00 (10.5)	11.00 (6.25)	9.00 (9.00)
Stool consistency	5.71 (1.06)	5.50 (0.86)	5.69 (0.96)

LI, low current intensity EA; HI, high current intensity EA; LC, loperamide control; SD, standard deviation; weekly SBMs, spontaneous bowel movement per week.

**Table 2:** Outcomes.

	LI	HI	LC
*The proportion of normal defecation*			
Baseline	0.11 (0.03, 0.21)	0.09 (0.04, 0.16)	0.09 (0.00, 0.20)
Week 4 change from baseline	0.19 (0.17, 0.40)^*∗∗*^	0.10 (0.06, 0.29)	0.15 (0.02, 0.37)^*∗∗*^
Week 8 change from baseline	0.28 (0.23, 0.56)^*∗∗*^	0.18 (0.11, 0.36)^*∗∗*^	0.16 (−0.03, 0.36)

*Weekly SBMs*			
Baseline	11.48 (10.54, 16.66)	13.60 (9.03, 14.51)	12.00 (8.83, 16.43)
Week 2 change from baseline	−1.95 (−3.34, −0.56)^*∗*^	−1.11 (−2.41, 0.20)^*∗*^	−2.72 (−4.21, −1.22)
Week 4 change from baseline	−2.02 (−3.64, −0.40)^*∗*^	−1.88 (−3.39, −0.36)^*∗∗*^	−3.56 (−5.30,−1.83)^*∗∗*^
Week 8 change from baseline	−2.77 (−4.00, −1.54)^*∗∗*^	−2.46 (−3.62, −1.3)^*∗∗*^	−2.51 (−3.83, −1.18)^*∗*^

*Stool consistency*			
Baseline	5.47 (5.22, 5.71)	5.51 (5.30, 5.73)	5.58 (4.65, 5.95)
Week 2 change from baseline	−0.89 (−1.24, −0.54)^*∗∗*#^	−0.37 (−0.71, −0.04)^*∗∗*#^	−1.16 (−1.55, −0.78)^*∗∗*^
Week 4 change from baseline	−0.86 (−1.24, −0.48)^*∗∗*^	−0.37 (−0.76, −0.01)^*∗∗*^	−1.21 (−1.62, −0.79)^*∗∗*^
Week 8 change from baseline	−0.91 (−1.22, −0.60)^*∗∗*^	−0.59 (−0.89, −0.30)^*∗∗*^	−0.74 (−1.08, −0.40)^*∗∗*^

Values are expressed as mean (95%CI). ^#^*P* < 0.05 and ^##^*P* < 0.01 vs. LC group. ^*∗*^*P* < 0.05 and ^*∗∗*^*P* < 0.01 vs. baseline. *P* values were for the comparison among the three groups and were calculated with the analysis of covariance (ANCOVA), except for the comparison of the baseline values, which used an analysis of variance (ANOVA). (1) The proportion of normal defecation defined as the proportion of a daily stool frequency ≤3 times and stool consistency to be type 4. (2) SBMs denote spontaneous bowel movements. (3) Stool consistency was assessed with the use of the 7-point Bristol Stool Form Scale (BSFS): 1 indicates separate, hard lumps, like nuts (hard to pass); 2 sausage-shaped but lumpy; 3 like a sausage but with cracks on the surface; 4 like a sausage or snake, smooth, and soft; 5 soft blobs with clear-cut edges (passed easily); 6 fluffy pieces with ragged edges or a mushy stool; and 7 watery, not solid pieces (entirely liquid).

**Table 3:** SF-36 scores.

SF-36	LI	HI	LC
*Physical Functioning*			
Baseline	92.81 (88.47, 97.16)	92.50 (87.85, 97.15)	92.50 (87.35, 97.65)
Change from baseline	−0.63(−4.12, 2.87)	1.75(−0.56, 4.06)	−5.31 (−19.76, 9.13)

*Role Physical*			
Baseline	70.31 (49.55, 91.08)	73.75 (54.22, 93.28)	85.94 (72.21, 99.67)
Change from baseline	3.13 (−21.13, 27.39)	7.50 (−5.70, 20.70)	−1.56 (−23.03, 19.90)

*Bodily Pain*			
Baseline	69.25 (57.88, 80.62)	67.70 (58.50, 76.90)	81.63 (74.16, 89.09)
Change from baseline	11.50(−0.05, 23.05)	0.50 (−7.32, 8.32)	−4.38 (−15.21, 6.47)

*General Health*			
Baseline	48.13 (38.21, 58.04)	57.50 (49.78, 65.22)	60.00 (49.94, 70.06)
Change from baseline	14.37 (5.20, 23.55)^*∗∗*^	8.50 (3.02, 13.98)^*∗∗*^	−0.62 (−14.13, 12.89)

*Vitality*			
Baseline	68.75 (59.50, 78.01)	71.25 (62.47, 80.03)	66.25 (58.44, 74.06)
Change from baseline	7.50 (1.35, 13.65)^*∗*^	3.25 (−1.69, 8.19)	0.94 (−13.50, 15.37)

*Social Functioning*			
Baseline	80.47 (67.38, 93.55)	84.38 (75.70, 93.05)	82.81 (74.79, 90.83)
Change from baseline	5.47 (−1.40, 12.33)	1.88 (−4.50, 8.25)	−3.91 (−16.48, 8.67)

*Role-Emotional*			
Baseline	70.83 (49.45, 92.22)	65.00 (44.46, 85.54)	77.08 (56.86, 97.30)
Change from baseline	6.25 (−16.42, 28.92)	13.33 (−5.19, 31.86)	−2.08 (−17.25, 13.08)

*Mental Health*			
Baseline	72.00 (62.82, 81.18)	76.80 (68.87, 84.73)	76.75 (69.91, 83.59)
Change from baseline	9.00 (2.02, 15.98)^*∗*^	3.60 (−2.71, 9.91)	−5.75 (−18.03, 6.53)

Values are expressed as mean (95% CI); ^*∗*^*P* < 0.05 and ^*∗∗*^*P* < 0.01 vs. baseline. *P* values were for the comparison among the three groups and were calculated with the analysis of covariance (ANCOVA), except for the comparison of the baseline values, which used an analysis of variance (ANOVA).

**Table 4:** SAS and SDS scores.

	LI	HI	LC
*SAS scores*			
Baseline	31.63 (27.08, 36.17)	31.85 (28.26, 35.44)	32.47 (28.26, 36.67)
Week 2 change from baseline	−3.56 (−7.55, 0.43)	−3.65 (−5.34, −1.96)	0.00 (−2.87, 2.87)
Week 4 change from baseline	−6.22 (−8.98,−3.46)^*∗∗*#^	−2.95 (−0.20, −5.70)^*∗*^	0.40 (−2.68, 3.48)

*SDS scores*			
Baseline	41.54 (37.03, 46.06)	38.81 (33.26, 44.37)	37.89 (33.40, 42.38)
Week 2 change from baseline	−5.00 (−9.71, −0.29)	−2.63 (−4.30, −0.95	−1.80 (−6.42, 2.82)
Week 4 change from baseline	−5.34 (−8.13, −2.54)^*∗∗*#^	−0.89 (−3.67,1.89)	1.09 (−2.01,4.19)

Values are expressed as mean (95% CI). ^#^*P* < 0.05 and ^##^*P* < 0.01 vs. LC group. ^*∗*^*P* < 0.05 and ^*∗∗*^*P* < 0.01 vs. baseline.

## Data Availability

The data supporting the research results can be obtained from the corresponding author.
